# Climber, mediator, and marathoner: narrative inquiry of career motivation changes of pre-service CSL teachers throughout teaching practicum

**DOI:** 10.3389/fpsyg.2024.1319507

**Published:** 2024-06-07

**Authors:** Rubing Zhou, Tianmiao Wang, Hanzhao Lin

**Affiliations:** ^1^School of Chinese as a Second Language, Faculty of Humanities, Peking University, Beijing, China; ^2^Research Institute of International Chinese Language Education, Beijing Language and Culture University, Beijing, China

**Keywords:** CSL teachers, career motivation, teaching practicum, narrative inquiry, metaphor

## Abstract

**Introduction:**

The growing global demand for foreign language learning contrasts sharply with the shortage of second language teachers. In Chinese as a second language (CSL) education, although the number of pre-service CSL teachers is increasing, few continue in the profession after completing their teacher education courses.

**Methods:**

To investigate the reasons behind this trend, this longitudinal narrative inquiry examined the career motivations of three participants during their teaching practicum. The study focused on identifying key narrative clues based on metaphors emerging from their narratives.

**Results:**

The research found that the participants’ career motivations were influenced by their teaching practice and experienced various dynamic changes. Key motivational factors included self-efficacy and intrinsic self-fulfillment, with a notable influence from the unique cross-cultural motivation associated with second language teaching.

**Discussion:**

The study underscores the significant role of narratives and metaphors in understanding changes in teacher career motivations. It suggests that enhancing self-efficacy and intrinsic motivation, alongside recognizing cross-cultural motivations, could be crucial in addressing the retention issues among pre-service CSL teachers.

## 1 Introduction

Teachers’ career motivation (CM) generally refers to a series of motivational factors that drive teachers to engage in or leave the teaching profession (Kelchtermans, 1993; Alexander, 2008). This topic has attracted scholarly attention in teacher research area over the past decades. Teachers’ career motivation not only reflects individual’s identification with and evaluation of the teaching profession, but also influences teachers’ enthusiasm and pursuit of the teaching profession, constituting the internal driving force of teachers’ professional development (Han and Yin, 2014; Hiver et al., 2018; Irnidayanti et al., 2020). The study of changes of teachers’ career motivation is especially pertinent and significant against the backdrop of the growing concern over the issue of teacher retention and a worldwide shortage of L2 teachers (Swanson and Mason, 2018; Dos Santos, 2022), because CM could be the starting point of teachers’ motivation and directly affects the stability of the teaching forces (Kissau et al., 2019; Zhang et al., 2020). In contrast with a large body of existing literature that focuses on ESL teachers’ career motivation, there is a remarkable lack of research on the career motivation of Chinese as a second language (CSL) teacher (Zhang et al., 2020).

The expansion of foreign language education has spurred a corresponding increase in the demand for Chinese as a second language (CSL) or a foreign language. Reports from the 2022 International Chinese Education Conference indicate that over 30 million individuals worldwide are engaged in learning Chinese in these capacities. However, this growth in demand for Chinese language education globally is met with a notable challenge: the instability of the CSL teacher workforce. Despite the burgeoning demand, the supply of qualified CSL teachers remains inadequate. According to the 2018 annual development report of Confucius Institutes, the worldwide count of full-time and part-time Chinese language teachers stood at a mere 47,000 by the end of 2018. CSL teachers are fundamental to the delivery of Chinese language education. Yet, they encounter a multitude of challenges, particularly those teaching abroad who face significant cross-cultural hurdles. This phenomenon has led to CSL education being perceived as “overwhelmingly a matter of Chinese [teachers] teaching Chinese to Chinese” (Scrimgeour, 2014, p. 154).

The Master of Teaching Chinese to Speakers of Other Languages (MTCSOL) program serves as a pivotal resource for training pre-service international Chinese language teachers. Despite the proliferation of MTCSOL programs and graduates, the issue of low employment rates for these graduates persists. Research conducted by Wang and Zhang (2021) underscores this, indicating that only a fraction of MTCSOL graduates secure employment as CSL teachers. While the attrition of pre-service international Chinese language teachers has been extensively studied, there remains a gap in understanding the underlying dynamics leading to their departure from the field of international Chinese education.

Thorough investigation into the lived experiences of CSL teachers within the realm of teacher motivation research is urgently needed as the representation of this group of teachers is largely understudied, which requires a detailed examination of their unique challenges and contributions, as well as their motivations, experiences, and subsequent retention within the profession. This examination, often with the assistance of qualitative research to capture individual subjectivity and experiences, has the potential to reveal crucial dimensions and add depth for understanding CSL/CFL teachers’ role and impact within the language teaching and learning field (Moloney and Wang, 2016; Wang and Mason, 2023).

In light of this, our research adopts a narrative methodology to explore the evolving career motivations of three prospective international Chinese language teachers throughout their teaching experiences. Through longitudinal observation and in-depth interviews as well as narratives obtained through diverse sources, we aim to elucidate their journey. Additionally, given humans’ inclination to use metaphors in recounting and comprehending life events, “they communicate knowing of one phenomenon through the lens of another, ultimately shaping not only an individual’s practical knowledge (knowing), but his or her actions (doing) and his or her identity (being) as well” (Craig, 2018, p. 301). Therefore, we will focus on identifying and analyzing the metaphors embedded within the participants’ narratives, in order to unearth insights into the educational values conveyed through them, and to delve into the dimensions of participant’s “knowing, doing, and being” (Craig, 2018, p. 300). This integrative approach will facilitate a nuanced understanding of the factors influencing their shifts in career motivations and professional decision-making processes.

## 2 Literature review

This section provides the theoretical underpinnings of the current study, concentrating on three aspects: (1) teachers’ career motivation and its influencing factors; (2) narrative inquiry and teacher motivation research; and (3) metaphors that teachers live by.

### 2.1 Teachers’ career motivation and its influencing factors

Early studies mainly divided teachers’ career motivation into intrinsic motivation, extrinsic motivation and altruistic motivation (Kyriacou and Coulthard, 2000; Dörnyei and Ushioda, 2010). There is a lack of precision in operational definitions, because early studies are not theoretically driven, resulting in overlapping classifications across studies. For example, the desire to work with children is often designated as an intrinsic motivation and also an altruistic motivation (Watt and Richardson, 2007). The applied research based on this structure mostly relies on self-compiled questionnaires as research tools, and the research results are mixed (Young, 1995; Hammond, 2002; Kyriacou et al., 2003).

The gradual development of motivation theories in psychology has promoted research on teachers’ career motivation. Expectation-value theory proposed by Atkinson (1957) is arguably one of the most influential theories in motivation psychology, which forms the theoretical basis for studies in teachers’ career motivation (Watt and Richardson, 2012; Eder et al., 2020). It holds that an individual’s belief in the successful completion of a task and their judgment of the task’s value directly affect their choice, persistence and performance (Eccles, 1983; Wigfield, 1994; Wigfield and Eccles, 2000). Following this line of logic, Watt and Richardson (2007) put forward the framework of “Factors Influencing Teaching Choice” (FIT-Choice), representing an important application of expectancy-value theory particular to the study of teachers’ career motivation. This framework expands the factors afforded by Expectancy-value model by incorporating the theory of social cognitive career theory (Bandura, 1986) and the fruits in teacher education research. To put it briefly, FIT-Choice mainly consists of six aspects - (a) career perceptions (task demand and task return); (b) self-efficacy (perceived teaching abilities); (c) intrinsic value; (d) personal utility value; (e) social utility value; (f) fallback career (Richardson and Watt, 2005; Richardson and Watt, 2006; Watt and Richardson, 2007).

Fit-Choice set a milestone for the study of teachers’ career motivation, because it helps solve the problem of lacking proper theoretical foundation in the study of teachers’ career motivation for a long time (Watt and Richardson, 2012). Since then, researchers around the world have investigated the career motivation of different groups of teachers based on this framework, and the results exhibit commonalities and effectiveness of the model. There are fewer studies on the career motivation of language teachers and most of them focus on ESL teachers (Zhao, 2008; Erkaya, 2013; Kissau et al., 2019; Zheng and Huan, 2022). While the results of research on the career motivation of L2 teachers are similar to those on general teachers based on the FIT-Choice framework (Karavas, 2010; Shih, 2016; Zhang et al., 2020), the difference in their career motivation between L2 teachers and general teachers mainly lies in cross-cultural factors. In other words, L2 teacher’s career motivation boasts unique cross-cultural factors, including interests in the social status or value of a specific L2 and the opportunity to experience the culture of developed countries and so on (Hettiarachchi, 2013; Hiver et al., 2018; Zhang et al., 2020).

Teachers’ career motivation would be affected by social factors (Watt and Richardson, 2007). With the worldwide application and validation of the Fit-Choice framework, Watt and Richardson (2012) further claimed that the differences in findings shall be attributed to the differences in social, cultural and economic development levels among different regions. Other factors that affect teachers’ career motivation include teaching practicum (Hiver et al., 2018), subject specialization (Akar, 2012), significant others (Bandura, 1986; Siwatu, 2007), educational resources and policies (Johnson and Birkeland, 2003), and demographic variables such as gender and age (Jantzen, 1981; Book and Freeman, 1986). Noteworthily, teaching practicum is widely considered as an important factor affecting pre-service teachers’ career motivation (Dörnyei and Ushioda, 2010; Topkaya and Uztosun, 2012; Zhang et al., 2020), because teaching practicums can furnish teachers with different views and deepen their understandings of the teaching profession (Sasson and Malkinson, 2021). These insights pertain to the differences between career perceptions of pre-service teachers and in-service teachers (Cortés, 2016), and of novice teachers and experienced teachers (Meristo and Eisenschmidt, 2014). For pre-service teachers, teaching practicum is especially influential in that the mismatch between hands-on experiences and their desired beliefs and values may weaken their career motivation and lead to distancing them from the teaching profession (Watt and Richardson, 2008).

### 2.2 Narrative inquiry and teacher motivation research

Being both mundane and powerful, story is able to reflect, express, inspire, astonish, create, etc. It is an essential avenue for accessing and understanding experiences (Xu and Connelly, 2010). Connelly and Clandinin (1990) underscore that humans are inherently storytelling beings, living and understanding their lives through the articulation and interpretation of stories, for “a person is, at once, engaged in living, telling, retelling, and reliving stories” (p. 4). This understandings positions stories at the heart of educational experiences, viewing educators and learners alike as active participants in storytelling and story-interpreting processes (Connelly and Clandinin, 1990). Meanwhile, experience is revealed in narrative form (Craig et al., 2018). As Connelly and Clandinin (1990) point out, “narrative is both phenomenon and method” (p. 2). Clandinin and Connelly (2000) contend that narratives offer a means of sensemaking, enabling individuals to articulate and contextualize their experiences within personal and cultural frameworks. Through narrative inquiry, researchers rely on relational co-construction of knowledge and embark on a journey alongside participants, traversing temporal, social, and spatial dimensions to uncover the rich, storied landscapes of educational experiences (Craig et al., 2018). This approach not only acknowledges but also celebrates the complexity and diversity inherent in educational settings.

Additionally, narrative research enables the examination of diverse viewpoints and opinions, thereby amplifying narratives that are often marginalized or ignored (Riessman, 2008). In other words, narratives can be function as agents of empowerment. By owning and sharing their stories, teachers assert their narrative authority, challenging dominant discourses and redefining the educational landscape. This empowerment through narratives fosters a culture of collaboration, innovation, and resilience, encouraging educators to reimagine their practices and engage with their communities in meaningful and impactful ways (Olson and Craig, 2005).

Narrative inquiry offers a platform for diverse voices, while also providing a reflective space for teachers to gain greater insight and empathy. Through narrative inquiry, educators can introspect their practices, beliefs, and experiences, fostering a deeper comprehension of their professional selves and motivations, which is conducive to sustainable professional development. Hence, narrative inquiry emerges as a useful and productive approach in the examination of teachers who teach Chinese as a second language or foreign language by paying attention to their stories, narratives, and experiences. For example, Wang (2022) offers an insightful examination into the intricate process and shifts of professional identity formation among pre-service Chinese language instructors in overseas educational settings, thereby illuminating the nuanced and complex factors influencing their decisions to exit the profession especially amidst a progressively intensifying and challenging global political landscape. Apart from native CSL teachers, Wang and Mason’s (2023) narrative exploration, based on a case study design, underscores the crucial role of intricate dynamics within teaching practicum and the continuous identity construction and negotiation by novice non-native CSL instructors across psychological, behavioral, and relational domains, showcasing an ethical and productive collaboration between native and nonnative CSL teachers. This exploration illuminates the diverse path traversed by non-native individuals who aspire to become Chinese language educators, emphasizing the significance of comprehending and addressing the unique challenges and opportunities they encounter to foster more inclusive and supportive professional environments for novice non-native language teachers and students against the backdrop of native-speakerism. Furthermore, Moloney and Wang’s (2016) utilized a dual narrative self-study approach to delve into the motivational substrates underpinning the career trajectories of both native and non-native CSL educators, thus accentuating the divergent narratives and issues of identity and power that shape their professional practices and pathways and calling for establishing de-centralized communities with a note of post-modernism. This narrative inquiry, to some extent, finds resonance in the work of Wang et al. (2013), where they scrutinized the pivotal nexus between CSL teacher knowledge and international curricular paradigms of teacher education programs of Chinese as a foreign language in China and Australia through comparative and interpretive lens, advocating for the refinement and internalization of teacher education initiatives and/or programs to align with the exigencies of a globalized educational landscape and to develop teachers’ situated manoeuvre and adaptation. When it comes to concrete professional practices, Bao et al. (2020) conducted an investigation into the experiences of five CAL teachers in employing teaching materials through the perspective and focus of teacher agency in shaping their professional praxis, thereby providing a comprehensive analytical framework to apprehend the interplay between individual agency and contexts. They found that teachers’ beliefs, teacher identity, and relationships within their community are key factors in the exertion of teacher agency in teaching practices, elucidating the manifold challenges encountered by practitioners and the adaptive strategies deployed to navigate the complexities inherent in their professional milieu, so that professional practices and multilingualism can be sustained.

These scholarly work are illuminating in some ways, for they center around CSL teachers and demonstrate the complex and dynamic interplay between teacher’s agency and situational conditions which spans across the macro, meso, and micro levels - from socio-geopolitical atmosphere and contexts, teacher education program’s curriculum and policy, to individual teachers as professionals - where CSL teachers work and live. Besides, most of them adopt narrative inquiry, supporting the usefulness of this research method in CSL teacher research, and they involve different types of Chinese as a second/foreign/additional language teachers, including those who study abroad at overseas institutions, novice and experienced non-native teachers and so on, giving a voice and attention to the not so ‘visible’ group of teachers, as compared with English teachers that are heavily discussed in the literature. Although these research provide solid bases and valuable insights for the current study, many of the settings of previous explorations are arguably homogeneous. Therefore, building on previous literature, this study supplements the narratives and experiences of native Chinese pre-service teachers immersed in overseas teaching practicum where different mobility and boundaries meet.

### 2.3 Metaphors that teachers live by

Metaphors constitute a crucial element of human language and cognition, enabling individuals to comprehend and communicate intricate concepts by relating them to more familiar notions. Lakoff and Johnson (1980) revolutionized the understanding of metaphor in their seminal work, “Metaphors We Live By,” arguing that metaphors are not just a matter of language but of thought and reason. Metaphors “make experience coherent” as well as structure and connect our perceptions, actions, and understanding of the world, embedding themselves in our everyday language and thought processes, (Lakoff and Johnson, 1980, p. 156). When conceptualizing teacher ‘personal practical knowledge’ as a holistic notion of classroom ‘images’, Clandinin (1985) admits that “images linked to experience in this narrative sense assume a metaphorical quality” (p. 380). This characterization echoes Craig’s (2018) discussion that “narrative inquiry promotes the identification and sense making of metaphors characterizing teachers’ experiences” (p. 302). These demonstrate that, in its essence, narratives and metaphors are intimately linked and closely associated.

More specifically, teacher metaphors generally refer to the conceptual and linguistic metaphors that educators use to describe, understand, and navigate their roles, practices, and the broader educational landscapes. That is, a teacher metaphor is an expressive tool that teachers use to compare their roles, responsibilities, and the educational process to familiar concepts, objects, or experiences, thereby offering insights into their perceptions of teaching and learning (Oxford et al., 1998). For example, Saban et al. (2007) emphasized the importance of examining metaphors used by teachers in order to reveal their understandings of teaching and learning. By utilizing metaphorical expressions like “teacher as gardener” or “teaching as journey,” educators communicate their viewpoints on their roles, their relationships with students, and their educational beliefs. These figurative expressions provide important insights into the implicit theories and cognitive frameworks that guide teachers’ actions and decisions in the classroom (Saban et al., 2007). Similarly, Kagan (1992) investigated the metaphors employed by teachers to characterize their experiences of curriculum change. Through qualitative analysis of interviews and reflective journals, Kagan identified metaphors such as “journey,” “battle,” and “dance,” each conveying distinct attitudes towards the process of curriculum reform. By exploring the metaphors held by teachers, Kagan shed light on the emotional and cognitive dimensions of curriculum change, highlighting the importance of attending to teachers’ subjective experiences in educational reform efforts.

Metaphors can be classified into ‘stock metaphors and novel metaphors’ and ‘emergent metaphors and ascribed metaphors’ (Craig, 2018, p. 302), among which novel and emergent metaphors are arguably more desirable to understand and represent the emic views held by educators in their own and creative voice or, put differently, presented in their terms from their perspective (Craig et al., 2017). Metaphors “bridge experience and meaning” (Craig, 2018, p. 310). To capture the diverse experiences and meanings in teaching and teacher education, Craig (2018) neatly summarizes and conceptualizes the fundamental functions and qualities of metaphors as ‘knowing, doing and being’ through elaborating on five metaphors emerged from narrative exemplars. Finally, Craig (2018) advocates that different kinds of metaphors “constitute promising catalysts of change in teaching and teacher education and in life itself” (p. 310), ending with a positive note and hope.

In summary, metaphors serve as a potent means of understanding the multifaceted and complex nature of teaching and learning in different contexts as discussed above. As with narratives, metaphors could carry different purposes, realize varying effects, and capture and construct meanings that might otherwise be inaccessible or elusive. By examining the metaphors that teachers live by, the education community can uncover the underlying beliefs, values, and assumptions that shape teaching practices. Furthermore, metaphors facilitate a reflective and critical examination of one’s teaching identity and approach, encouraging ongoing professional development and innovation in educational practices (Saban, 2006). Through the study and use of teacher metaphors, educators can not only articulate their own experiences and understandings, but also contribute to the broader dialogue on effective teaching and meaningful learning as seeds for pedagogical innovations and facilitating communication among educators, policymakers, and the wider community (Bullough, 1991; Tobin and Tippins, 1996). Therefore, the integration of teacher metaphors with narrative inquiry, which affords situated contexts and experiences, holds educational value and warrants further exploration in its own right.

## 3 Methodology

Methodologically speaking, most of the surveys on teachers’ career motivation are quantitative studies, which captures no more than the state at a static point. Although quantitative methods can provide validity and replicability, they lack the descriptive details and depth that qualitative research provides (Onwuegbuzie and Leech, 2005). Because teachers’ career motivation is dynamic and complex, some researchers believe that over-reliance on quantitative methods in the study of teachers’ career motivation limits our understanding of the contents and functioning of teachers’ motivation (Parr et al., 2021). Furthermore, due to the late start of the research on CSL teachers’ career motivation, it calls for more systematic investigation. Although the role of professional identity in maintaining the career motivation of pre-service CSL teachers has been highlighted through qualitative research (Wang and Zhang, 2021), gaps remain and research on the career motivation of CSL teachers needs to be furthered (Zhang et al., 2020), especially with the help of narrative inquiry to gain more in-depth insights by focusing on pre-service CSL teacher’s teaching practicum stage which directly affects their career choice afterwards.

### 3.1 Studying teachers’ career motivation narratively

Narrative research presents, reconstructs, and interprets teachers’ experiences in teaching, learning, and research within a three-dimensional narrative space of time, characters and society, and location or context (Clandinin and Caine, 2008; Craig, 2011). Through this exploration of teachers’ inner worlds, narrative research aims to reveal their essence and significance. Thus, educational narratives can better help us investigate the dynamic and elusive professional motivations of teachers. In narrative research, researchers need to co-construct field texts with participants, interpretively record events and feelings, and then, after negotiation with participants, create a comprehensive narrative and research text positioning it within academic discourse (Clandinin and Connelly, 2000). Narrative research typically employs three analytical tools: ‘broadening’, ‘burrowing’, and ‘storying and retelling’ (Connelly and Clandinin, 1990). Recently, a fourth tool – ‘fictionalization’ - has been introduced to respect and further protect the privacy of participants’ narratives (Craig et al., 2018). This study provides an overall description of the experiences of three pre-service international Chinese language teachers in the educational practice process, in which the changes in their professional motivations naturally emerge and are extracted as they share their stories.

### 3.2 Research context and participants

This study is based on the teaching practicum of MTCSOL program. The exact teacher education program generally lasts for 2–3 years in different universities, and its courses generally consist of three indispensable parts: academic courses, teaching practicum, and M.A. thesis writing and defense. There are two main types of teaching practicum for students in MTCSOL: one is to apply for volunteer programs to teach Chinese abroad, and the other is to teach Chinese in universities and international schools in mainland China.

We adopted ‘purposive sampling’ to select six MTCSOL students among the candidates who were interested and willing to participate in this study. They were selected because (1) the participants had strong expressive and reflective abilities to tell stories that were related to research questions, offering rich and thought-provoking details and ideas, which renders the data more robust and is beneficial to subsequent data analysis and interpretation; (2) their experience could be categorized into three different types of teaching practicum that is characteristic of pre-service CSL teacher preparation, including teaching in overseas, domestic, and domestic as well as overseas contexts; as a result, there are two participants under each type of teaching practicum, providing a good arena for comparison and discussion. As part of a larger longitudinal project, the current paper only reports three participants, Zoe, Tina, and Gimi (all pseudonyms; see Table 1 below for demographic information), mainly because their stories represent three typical changes in CSL pre-service teachers’ career motivation throughout teaching practicum and these cases showcase differing career choices. The three kinds of career choices are basically in line with the three main paths of employment for MTCSOL graduates (Hu and Wang, 2020).

**Table 1:** Participants’ background information.

Name	Gender	Age	Type of teaching practicum	Length of teaching practicum	Career choice
Zoe	Female	28	Abroad (USA)	1 year	Pursuing a Ph.D.
Tina	Female	29	China (International School + Domestic University)	1 year and 3 months	Primary school Chinese teacher
Gimi	Male	28	China (International School) + Abroad (UK)	8 months	CSL teacher in a Chinese university

### 3.3 Data collection and construction of field texts

In narrative research, researchers need to construct field texts with participants, make explanatory records of events and feelings in researcher’s memos, and then create a holistic narrative text after consultation with participants and position it within academic dialogue (Caine et al., 2013). The field texts in this study primarily originated from (1) participant observation; (2) in-depth interviews; (3) small stories; (4) physical materials; and (5) multimedia materials. All materials and data were obtained with the explicit and informed consent of the participants.

#### 3.3.1 Participant observation

The creation of field texts spanned 3 years, during which the researchers engaged in long-term participant observation of the participants’ learning and work lives. This involved either living with some of the research subjects, attending a course together for a semester, participating in training sessions for over a month, or maintaining close contact over an extended period. Throughout this process, the researchers entered the exploratory space, engaging in social interactions with the research participants and the research environment as insiders, and writing field notes. By observing the interactions between the research participants and other social individuals, researchers were able to more directly acquire evidence from the real three-dimensional space.

#### 3.3.2 In-depth interviews

We had numerous informal communication and two rounds of one-on-one formal in-depth semi-structured interviews, each of which lasted for 60–125 min. The first round of interviews mainly investigated the demographic information of participants, the motivation of choosing MTCSOL to be their major, the type of teaching practicum that they engaged in and relevant information, the stories of teaching practicum and its impact, the current job information and motivation for choosing them, etc. Special attention was paid to critical incidents in the teaching practicum and participants’ perceptions and articulation of their motivation changes in response to their teaching experiences. In the second round of interviews, after the preliminary data analysis, key information and pivotal nodes in the first round of interviews were followed up and supplemented.

#### 3.3.3 Small stories

Small stories, also known as narratives-in-interaction, are characterized by atypicality and non-normativity. These personalized and situational small stories can coexist with grand narratives and often lie on the margins of overarching narratives that cannot be explained (Georgakopoulou, 2006). The researchers maintained continuous contact with all participants and engaged in multiple short informal conversations around the research questions. These narratives-in-interaction occurred in a very relaxed atmosphere, where participants were willing to share their inner feelings, thoughts, and experiences, demonstrating a high degree of authenticity.

#### 3.3.4 Physical materials

Physical materials primarily consisted of proof materials voluntarily submitted by participants for the researchers’ review. These materials included teaching designs, work summaries, internship certificates, award certificates, student comments, thank-you letters from parents, reflective journals, master’s theses, etc., from participants during their teaching practices. Most of these physical materials were photographed by the researchers with the participants’ consent, with key information mosaic-ed for archiving. Physical materials helped researchers verify the authenticity of participants’ experiential stories to some extent, thus avoiding exaggeration or falsity.

#### 3.3.5 Multimedia materials

Multimedia materials mainly referred to publicly available data posted by participants on their social platforms, including reflections and certificate sharing during their learning and work periods, communication photos with students and colleagues, etc. Some publicly available data were processed to remove privacy information with participants’ consent. These multimedia materials not only helped researchers organize participants’ personal experiences according to the timeline, supplementing and corroborating their narratives but also stimulated participants to recall their experiences, enriching their narrative accounts.

Overall, narrative materials obtained from in-depth interviews and researchers’ participant observations were the main sources of field texts. Narratives-in-interaction, physical materials, multimedia materials, etc., were used to assist in refining the field texts. Researchers triangulated these data to vividly reconstruct the stories of participants’ changes in professional motivations during their teaching practices.

### 3.4 Narrative analysis

Narrative analysis of this study can be divided into two main stages: (1) to determine the narrative clues and establish narrative accounts; (2) to quest for the resonance and differences between different participants’ stories through the cross-case analysis of participants’ narrative accounts, and to analyze and discuss findings against theoretical underpinnings.

In the first phase, the participants’ field texts were placed in a three-dimensional narrative space. Then, we extracted the key events, characters, emotions and places that affect the transformation of their stories. The specific steps are shown in Table. 2.

**Table 2:** Analysis steps in the first phase.

1	Transcribe conversation records and interviews into texts for narrative coding to identify and code key narrative threads
2	The observation records of the researchers and the materials submitted by the participants were placed in the field text for triangulation
3	Extract the theme of the story narrated by the participants, sort it according to the three-dimensional space, and initially write the narrative accounts of the participants
4	Communicate with participants about their narrative accounts, seek out the resonance narrative clues, and revise the narrative account based on participants’ and coauthor’s feedback and iterative analysis

In sum, in the narrative analysis of this paper, we rely on the theoretical framework of Fit-Choice theory and the findings of research on second language teacher motivation as our theoretical foundation. We employ four narrative analysis tools: broadening, burrowing, storying and restorying, and fictionalization. These analytical tools are used to interpretively construct the development of participants’ professional motivations based on the meanings attributed to their experiences. When writing narrative accounts, researchers strive to directly utilize participants’ own expressions as much as possible for the sake of trustworthiness and narrative authenticity, preserving participants’ idiomatic and local expressions on key narrative threads.

## 4 Narratives of experiences

The results of this study are the narrative accounts constructed on the basis of participants’ experiences and stories holistically. More importantly, emergent and novel teacher metaphors are drawn from the field texts to form the important basis of analysis and comparison. Specifically, metaphors of persistent climber, transformed mediator and practical marathoner are discussed.

### 4.1 Zoe: a persistent climber

Zoe’s undergraduate major had nothing to do with Chinese education, but she chose to apply for MTCSOL program, because she was moved by her friend’s enthusiasm for CSL education. “I think this major is very interesting... When I think I can share language and culture abroad, my blood surges!” Zoe believes that her Chinese language learning performance has been excellent from childhood to adulthood. When talking about her application to the MTCSOL program, she expressed great confidence, stating, “I should not be much different from students in this major... I am very capable, and I easily passed the exam.”

#### 4.1.1 Early in the teaching practicum

After enrollment in the master degree, Zoe expressed her desire to apply for overseas teaching practice and emphasized her preference for going to the United States. This sparked considerable discussion in the class because everyone knew that internship opportunities in this country were very limited and the competition was intense. Despite lacking relevant teaching experience, confident Zoe still hoped to make adequate preparations:

"I want to experience what real teaching of Chinese as a foreign language is like. I previously went to Australia alone, staying in hostels and meeting people from different countries... I tried teaching them some simple Chinese, and they were very interested and had many cultural questions. I also found this kind of cross-cultural exchange to be particularly great! I want to go to the United States again, so I first applied for an online Chinese teaching part-time job in the United States. Although it was less than a month, that 'trial' was particularly successful, and the students gave me very high ratings... I am more confident about applying for an internship in the United States!"

Shortly afterward, Zoe successfully went to the United States. Despite encountering some difficulties, such as long working hours and inconvenient commuting, she worked hard to overcome them and achieved some successes at work. During the first few months of her teaching practice, she appeared very excited and happy:

"My colleagues and students all like my classes; they are very friendly to me and are very interested in Chinese characters and Chinese culture... My efforts have been recognized. I feel very accomplished! Most American colleagues have their own cars, but I don't have a driver's license, so I can only take the bus to work... The commute is very long, and my personal time is very limited. Although it's tough, overall, every day is fulfilling and joyful. My classes are going well, and parents praise me on Facebook."

#### 4.1.2 Midway into the teaching practicum

As the teaching practicum progressed, Zoe still believed that she was “suitable and will definitely become an CSL teacher,” but she encountered some setbacks in the teaching process. Zoe’s spoken English did not reach the level where she could comfortably communicate with American colleagues. She felt distressed during work meetings because of the abundance of professional terminology and fast speech pace. She could only understand the gist and was unwilling to actively share her experiences each time, reflecting that -

"I thought my English level was good, but now it seems that although teaching in class is not a problem, having in-depth communication with students and colleagues is more difficult... I always have a headache during routine meetings because their Chinese level is not very high either, and if I speak Chinese entirely, they also find it difficult to keep up. I also lack the confidence to communicate teaching experiences entirely in English... Being a teacher is not just about delivering a good lesson; it's also about understanding students and learning from colleagues."

Zoe has gained new insights into the profession of CSL teachers. She started studying English and taking teaching notes in the school library every week, continuously adjusting and improving her courses. Six months later, Zoe was able to communicate freely with her colleagues. During breaks, she also helped these local Chinese teachers improve their Chinese proficiency and teaching skills. Her efforts received unanimous praise, as she realized:

"There are always more solutions than difficulties, and my self-study effect is very good... On the surface, it seems like I am teaching them, but in reality, I am constantly comparing and learning in this process, reflecting on my teaching, and so on. It's like we are learning from each other... I received an outstanding teacher certificate from the school, but I don't think my lessons are already perfect. The certificate is everyone's recognition of me, indicating that my efforts have been seen and my achievements have been acknowledged, which also gives me more motivation."

#### 4.1.3 Later-stage of and after the teaching practicum

As students’ proficiency improved and the teaching content became more advanced, Zoe encountered new challenges several months later. Zoe, who has always been reflective, believes that her classroom teaching ability is good, but she faced difficulties in addressing some theoretical issues.

"I gradually realized that I may not be able to answer some questions from high-level students immediately, not because I cannot express myself in English... I need to look up materials after class to clarify things. I always thought I was a well-rounded student, especially in practical skills... but since I didn't major in this field before, my theoretical knowledge is not sufficient... I know a qualified teacher of Chinese as a foreign language must have both theoretical and practical abilities..."

Zoe reported her problems to her mentor in China. Through online guidance from her mentor, she initiated action research to analyze and solve the problems she encountered. With the guidance and assistance of her mentor, Zoe gradually regained her confidence. She firmly believes that she must stay in the field of international Chinese education and realizes the need to enrich her theoretical knowledge further. She decided to apply to become a doctoral student in the field of international Chinese education with the encouragement from her mentor and her growth stemming from the teaching practicum.

"My mentor encouraged me to find a thesis topic during teaching practice. She provided me with a lot of help and guidance. From not being able to understand some academic papers at all to being able to apply some theories to solve practical problems... I feel that my academic ability has improved, and I have developed an interest in research because I had a great experience during this process! I will definitely continue in this field, but I still have many shortcomings in theory... I want to further improve my professional abilities through pursuing a Ph.D. During this year of teaching practice, I feel like I am climbing a mountain. There are comfortable zones that allow me to climb smoothly, and there are steep and rugged areas that hone my skills... but I am constantly climbing upward and need to continue recharging myself through learning to keep going upward."

### 4.2 Tina: a transformed mediator

Since Tina’s undergraduate major is Chinese language and literature, she does not know much about MTCSOL. After weighing the success rate of different majors in the national postgraduate entrance examination, she chose to apply for MTCSOL with a practical extrinsic motivation, because “the professional study content of Chinese language and literature itself forms the basis of Chinese language teaching. I think I should not have any problems... The entrance exam content was really quite simple for me.”

#### 4.2.1 Early in the teaching practicum

With the advantage of her undergraduate background, Tina easily entered the MTCSOL education program and was very confident in completing her education plan smoothly. Although she did not have a deep understanding of the profession of CSL teacher, she believed that “any Chinese person can teach foreigners Chinese... There should not be much threshold for this profession.” Influenced by her family background, Tina was unable to intern abroad and conducted her first teaching practice domestically which is described below:

"Our school's training program stipulates that at least 6 months of teaching internship or corresponding teaching hours are required. I chose an international school affiliated with our school for teaching practice... Although this was my first formal Chinese as a second language teaching experience, there was a relatively complete internship process here... The atmosphere at the school was very good, with many students, mostly elementary school students... I taught relatively basic courses, and for the first few classes, there was a special internship mentor. I was basically an assistant, learning and observing more... The first month passed quite quickly and smoothly."

Before long, Tina began independent teaching. Without overseas experience, she engaged in friendly cross-cultural communication with her students. The pleasant teaching experience immersed Tina in the novelty of cross-cultural interaction and maintained a relatively high sense of self-efficacy.

"I feel it's more interesting than teaching Chinese to Chinese people. It feels quite simple... I quite enjoy it... I haven't been abroad before, and I haven't had much contact with foreigners... Students come from different countries, and every day brings new cultural experiences. The students are quite well-behaved, and the activities I arrange in class are quite active... They are young, so we mostly play games... The students really like me; they even brought me specialties from different countries, various small gifts... They wrote little cards for me, and when I translated them online, they were mostly praising me... Teaching foreigners feels quite simple."

#### 4.2.2 Midway into the teaching practicum

Entering the mid-term of the teaching practicum, the schedule of the teaching curriculum became tighter. As the courses progressed, Tina began to feel overwhelmed. Due to the unified curriculum requirements of the international school, Tina could not teach as freely as she wanted. Additionally, after losing the guidance of her internship mentor, Tina failed to find other ways to address the problems, leading to constant self-doubt.

"The initial courses were so simple. I didn't expect the teaching to become so difficult later... We only had guidance teachers at the beginning, and now I have to adjust by myself... I still need to meet the assessment requirements of the international school for intern teachers. With limited class time, adding too many interactive activities will prevent me from completing the teaching content required by the curriculum... If the course progress is slow, I can't just keep playing games, and I can't control the students... If I keep teaching from the textbook, the class will be very boring, just repeating drills every class, and the students won't like it... I really feel like I can't do it."

During that time, Tina behaved very irritable and emotionally agitated because she also encountered some cross-cultural conflicts. Tina’s class was a zero-basic class, and the students’ Chinese proficiency was very low. Her class started with teaching Chinese phonetics, and she could not teach entirely in Chinese. Tina realized that teaching foreigners was not as simple as she thought, and gradually blamed herself for the teaching difficulties, repeatedly saying, “I’m not capable enough,” “I cannot teach well.” Her frustration resulted from teaching experiences is demonstrated below, which negatively affects her self-efficacy as a teacher:

"It's completely different from how I felt when I wanted to teach Chinese to foreigners at the beginning... They don't understand, and I can't explain... It's my own lack of ability. I can't teach well... I can't teach them well... It's too difficult to communicate with them, I can't meet my emotional goals or my teaching goals... Maybe I'm just not suitable for teaching foreigners without any language foundation."

#### 4.2.3 Later-stage of and after the teaching practicum

The internship duration at the international school was only 3 months, but Tina, unwilling to give up, decided to give teaching practicum another try. After careful consideration, Tina applied for a teaching position at the international department of a Chinese normal university affiliated high school, starting her later-stage teaching practicum. The majority of students in this position were either Chinese descendants or mixed-race individuals who grew up in China, with relatively high proficiency in Chinese. Their main goal was to apply to foreign universities, and Tina’s responsibility mainly involved teaching courses related to Chinese culture.

“The course itself is quite simple; it’s just about telling stories. I collect some materials on Chinese cultural stories, make them into PowerPoint presentations, and deliver the content. Since this course does not involve exams or further education, the students mainly focus on preparing for the IELTS and TOEFL exams for overseas university applications... Although teaching this course is straightforward, I do not feel any sense of accomplishment... I do not need to prepare much for class later on, it’s just casual teaching because they do not pay attention anyway... My class is just for show, and what’s infuriating is that some students even play cards during class... If they do not care, why should I? I really feel it’s meaningless.”

In the final stages of the teaching practicum, Tina had completely given up on becoming a CSL teacher. The school’s lack of emphasis on this course made her doubt this profession. As the teaching practicum was nearing its end, Tina politely declined the school’s request for a high-paying contract renewal. Eventually, Tina chose to become an elementary school teacher in China because, despite her many frustrations during the teaching practicum, this challenging experience made her transition to applying for elementary school teaching positions relatively smooth.

"Throughout the teaching practicum, I have been reconciling my initial perceptions of this profession with the realities I encountered during the practicum... At first, I thought this profession was simple, with lots of recognition, low threshold, and high returns... but the reality turned out to be quite the opposite, so I had to constantly adjust my inner feelings... Being a CSL teacher is really not as meaningful as I thought. I've come to realize that now... Teaching Chinese to elementary school students is much simpler than teaching Chinese to foreigners, I think because I gained a lot of experience and growth from my previous teaching practicum... My teaching demonstration during the job interview received unanimous praise, which gave me a sense of accomplishment!"

### 4.3 Gimi: a practical marathoner

Gimi majored in CSL education as an undergraduate, because his father thought this major was “promising” and “could land a decent job” in the booming trend of “Chinese fever.” After entering school, Gimi posted a world map in his bedroom, declaring that he was determined to “see the outside world.”

#### 4.3.1 Early in the teaching practicum

After enrolling, Gimi aimed to undertake teaching practicum in Europe or America. Despite his major in the field, his lack of relevant teaching experience led to failure in his application. The setback of not being selected for overseas practicum deeply affected him, evident in his lack of confidence during our conversations:

"The competition for positions in Europe and America is too fierce and challenging. I didn't volunteer during my undergraduate years, so I lack the experience of teaching abroad. I didn't get selected even for a small European country in the first round of interviews... The second round for overseas volunteer positions will take another half year, and I don't have much confidence in myself... I think I'll start with an internship at an international school first, get a certificate, and earn credits."

Choosing to start his teaching practicum at an international school in China, Gimi was accompanied by three other classmates, all of whom would undergo the internship evaluation simultaneously after 3 months. At this point, Gimi’s self-efficacy was notably low, and he once expressed, “I cannot compete with them... I have no advantages.” The international school is a partner school of Gimi’s university, and each intern was assigned a mentor teacher. Gimi felt that his mentor teacher had been very helpful:

"Teacher L has always been nice and helped me solve problems... Initially, I was her teaching assistant, and she meticulously explained every aspect of teaching to me, knowing that I was anxious and lacked confidence, she kept encouraging me... Even after I started teaching independently, she would often guide me and reassure me that I could always come to her with any issues. My first solo class received high praise from her. I've always been afraid of comparing myself with other interns, but Teacher L has consistently encouraged and praised me, not blindly, but because she truly helped me progress... I feel my teaching is being recognized, and I'm striving to adjust it along with her guidance."

Three months later, with the help of his mentor teacher, Gimi regained his confidence, realized his self-worth, and received an excellent rating in the internship evaluation. “I did not expect this outcome, especially after being rejected before. Now I feel like I’m back on track!”

#### 4.3.2 Midway into the teaching practicum

Regaining his confidence, Gimi participated in the second round of interviews for overseas Chinese language teacher volunteers. This time, he was successfully dispatched to teach in the UK. Gimi’s teaching task was to conduct Chinese interest classes at a primary school in a small town in the UK. This position did not require high qualifications for Chinese teachers, nor were there strict requirements for curriculum content and progress. As a matter of fact, the previous instructors were not even graduates in international Chinese education-related majors. After a period of teaching, Gimi found that he achieved better teaching results than the previous teachers, which boosts his self-efficacy.

"Everyone feels that my teaching content and design are better, which gives me more confidence... The previous teachers here were not students from our major, and their teaching feedback was not very good... The courses I design here are mainly based on mainstream Chinese teaching materials in the UK... The response has been particularly good. Parents always write thank-you letters, saying that their children are becoming more interested in Chinese and even want to hire me as a private tutor."

During that time, Gimi often sent the researcher videos and activities that he organized during class, always with a smile on his face. Gimi believed that his excellent teaching effectiveness stemmed from the high standards of training for CSL teachers prior to their careers. He attributed this success to the academic and professional training he received, which laid a solid foundation for his role as an overseas volunteer. Gimi considers this profession to be highly specialized. At the same time, he believes that he is also a practical person who pays attention to the application of knowledge. Hence, his character traits are compatible with the professional demands in his views.

"In our MTCSOL program, the requirements are quite high. Since I study this major, so of course I perform better than them. It's all about applying what you've learned. Teaching a group of elementary school students, even though it's akin to an elective course, it's incredibly enjoyable. The teaching process is a breeze for me, as I can handle everything with ease. No problem at all! I even structured the elective course into different levels, making it more systematic as it progressed. And I received recognition as an outstanding teacher."

For nearly half a year, Gimi spoke excitedly about his classroom experiences, repeatedly referring to the job as “a piece of cake” and proudly displaying his certificates and letters of commendation.

#### 4.3.3 Later-stage of and after the teaching practicum

Gimi’s recount of his overseas teaching practice interview experience revealed a significant lack of confidence, contrasting sharply with his “bursting with confidence” demeanor in his position in the UK. His teaching practicum went very smoothly, and through this experience, he gained a better understanding of the CSL teaching profession and raised his expectations of himself as an international Chinese language teacher. A year went by quickly, and Gimi felt very reluctant to leave. At the same time, he hoped to make some “real contributions” to his students and Chinese language education in the UK in his final stage of teaching practicum.

"I'll never forget this experience. I've compiled my teaching designs to leave them for the next intern taking over my position... I'm very reluctant to leave these students. I can only do so much for them, but I'll try to pass on what I can, which counts as 'learning by doing,' right? I hope this interest course can become more systematic in the future, turning into a more formal curriculum and giving more people interested in Chinese language the opportunity to learn. That would be the most practical."

After returning to China, Gimi organized all his teaching materials and found research questions from them. He completed his thesis based on the practical problems he encountered in teaching. Gimi was determined to become a frontline international Chinese language teacher, likening the path of CSL teaching to a marathon, stating that he would continue to “learn by doing.”

"I'll definitely continue teaching Chinese. I've learned so much and done so much; I have to apply it, otherwise, it's wasted... To be honest, I still don't really know how to write a thesis. I've read a lot of them, and they're mostly abstract. My thesis is based on practicality... I prefer and am better at doing practical things, so I won't continue with a Ph.D. I chose a teaching position at a university, mainly teaching Chinese to international students, without having to do research. This is what I want. I believe teaching is a marathon. Only by being down-to-earth and learning by doing can I avoid stepping on air... This position allows me to realize my value more, and I'll continue to learn in practice and constantly apply what I've learned to become a better CSL teacher."

## 5 Serial interpretation and discussion

Through reading and organizing the narrative accounts of the participants, we found that their career motivations underwent dynamic changes, influencing their choices of three different career development paths. This result demonstrates the dynamism and situational characteristics of teacher career motivation, indicating that teacher career motivation will change with changes in the external environment. Zoe initially firmly believed that she was very suitable and would definitely become an CSL teacher. However, she gradually discovered many difficulties, and her lack of English proficiency and theoretical knowledge made it difficult for her to support further professional development, causing her to feel uneasy and doubtful. However, believing that there are always more solutions than difficulties, she constantly reflected on and improved herself, ultimately realizing that teaching is an upward climb, further enhancing her expectations of teaching. Tina, on the other hand, started off believing that teaching foreigners was very simple, but as the practice progressed, she gradually realized that the profession of CSL teacher was very different from her imagination. From doubting her own abilities to believing that her work was meaningless, her expectations of teaching collapsed gradually amid continuous coordination. Gimi, in complete contrast to Tina, although faced setbacks when applying for overseas practice, after being tempered by teaching practice, he, resiliently, not only regained confidence but also further enhanced his teaching ability and expectations of teaching in “this marathon.” The metaphors emerging from the narratives of the three participants serve as distilled representations of their career motivation changes and choices. Ultimately, the three participants symbolize three metaphors: ascending within the professional field through further studies (the Climber), departing from their major to become teachers of other types (the Coordinator), and working as full-time international Chinese language teachers within the field (the Marathoner).

After comprehending and analyzing the narratives and metaphors utilized by the participants, we proceed to deliberate on the five predominant themes that are consistently present as below: (1) the high driving effect of internal motivation; (2) cross-cultural motivation; (3) important influences of teaching practicum; (4) contribution of narrative inquiry to teacher motivation research; and (5) values of metaphors in narrative inquiry.

### 5.1 High driving effect of internal motivation

Through comparative analysis, we found both similarities and differences in the motivational factors influencing the career choices of the three participants. Teacher career motivation is characterized by complexity and dynamism. We discovered deep qualitative changes in the key narrative threads, where motivations persistently present in the participants’ accounts underwent shifts in their essence.

In this study, such qualitative changes primarily manifested as variations in intrinsic motivation, namely self-efficacy and intrinsic self-fulfillment values. Self-efficacy, or belief in one’s capabilities, refers to an individual’s perception of their current abilities in a specific activity (Eccles and Wigfield, 1995), in this study, indicating the participants’ perceived level of competence required to become international Chinese language teachers. Intrinsic value refers to the enjoyment derived from completing a task (Watt and Richardson, 2007), with intrinsic self-fulfillment value demonstrating that an individual’s efforts are recognized and respected by others, leading to inner satisfaction and joy. Zoe experienced fluctuations in her self-efficacy, deepening her understanding of the requirements of the international Chinese language teaching profession. Tina’s self-efficacy and intrinsic value gradually diminished, leading to a shift in her career perception after failed coordination attempts. Gimi’s self-efficacy underwent a curve from weakness to strength, achieving self-realization and solidifying his marathon-like career belief. We believe that these qualitative changes in intrinsic career motivation constitute the core driving force behind the participants’ career choices.

It appears that none of the three participants substantially, or at least explicitly, considered career rewards in their actual career choices, with Gimi even opting for a lower-paying job. This contrasts with the findings of Wang and Zhang (2021), whose research showed that the career choices of pre-service international Chinese language teachers were more influenced by external factors such as salary. Perhaps this is because our study unfolded through teaching practicum as an intermediary. This is in line with Bastick’s (2002) study on Jamaican teachers in which Bastick found that teachers with teaching practices experience were more influenced by intrinsic motivation and less affected by extrinsic motivation.

### 5.2 Cross-cultural motivation

The unique cross-cultural motivation of second language teachers includes factors related to cross-cultural experiences, such as the desire to learn foreign languages and cultures, an interest in cross-cultural communication, and frequency of interaction with foreigners. All three participants in our study displayed cross-cultural motivations in their career aspirations. Firstly, both Zoe and Gimi engaged in overseas teaching, expressing great excitement about their experiences abroad. Part of Zoe’s decision to major in international Chinese education was driven by her desire to teach abroad; she firmly expressed a desire to “experience authentic teaching of Chinese as a foreign language in the United States.” Gimi hung a world map on the wall in his dormitory to inspire himself to venture outside. He “has always wanted to explore the world outside, experience different cultures and lifestyles.” Therefore, even after his initial failure in applying for an overseas teaching position, he persisted in reapplying, driven by this desire to experience different cultures and lifestyles. Additionally, the cross-cultural exchanges experienced by Zoe and Gimi during their overseas teaching had a positive impact on their career choices. They both expressed enjoyment in interacting with local people, students, and colleagues, and gained insights in various aspects including culture, language, and friendships. In contrast, Tina’s motivation for cross-cultural communication had a reverse effect on her career choice as an CSL teacher. Initially fascinated by cross-cultural interactions, describing the experience as “interesting, with different cross-cultural experiences every day,” Tina later experienced cultural conflicts, feeling disappointed when realizing that her attempts to convey Chinese culture were not as simple as she had imagined, leading to a loss of confidence. It is worth noting that even after choosing a career as an elementary school teacher, Tina still retained an interest in experiencing cross-cultural life, stating that she “would still like to go abroad to experience teaching Chinese if given the opportunity.” Cross-cultural exchange motivations were present in the career motivations of all participants in this study, aligning with the findings of Zhang et al. (2020), which identified unique cross-cultural motivations in pre-service CSL teachers. However, in this study, cross-cultural motivations did not play a major role in driving the participants’ career choices.

### 5.3 Important influences of teaching practicum

The teaching practicum has a significant impact on the career motivation development of the three participants, particularly on the influence of their core driving motivations. As a critical component of teacher education, the impact of teaching practice on pre-service teachers’ self-efficacy has been widely recognized in academia (Dalioglu and Adiguzel, 2016; Sasson and Malkinson, 2021). Zoe, after completing her teaching tasks, experienced an increase and maintenance of self-efficacy: “The students gave me high ratings, I am very confident... I have teaching ability”; Gimi initially expressed doubts about his abilities after encountering setbacks in the early stages of teaching practice, but after diligently completing teaching tasks, his self-efficacy also significantly improved: “It’s a piece of cake”; whereas Tina, due to difficulties in fulfilling teaching tasks, gradually lost confidence: “It’s too hard... I cannot teach well.” These research findings also reflect the positive correlation between teaching practicum and teachers’ self-efficacy (Caprara et al., 2006; Holzberger et al., 2013).

Regarding intrinsic self-fulfillment motivation, teaching practicum exerted a significant reverse impact on Tina and Gimi’s intrinsic self-fulfillment motivation. Tina felt that she “could not achieve my teaching and emotional goals... I have no sense of achievement,” whereas Gimi “felt recognized... received many thank-you letters.” Zoe’s intrinsic value was to some extent realized during teaching practice, with relatively smooth fluctuations. Like Tina, pre-service teachers may have certain expectations for teaching practice: “Feeling that teaching Chinese to foreigners is very simple.” These prior expectations may underestimate the complexity of practical tasks (Atay, 2007), leading to damage to the self-realization value of pre-service teachers. Although conflicts between ideals and realities are common in pre-service teaching practice (Brown, 2006), the outcomes may vary. Faced with the demands of teaching practice, some pre-service teachers may enhance efficiency, confidence, and problem-solving abilities through various means (Tillema, 2000; Caires et al., 2009). Guided by mentors and continually reflecting and adjusting, both Gimi and Zoe ultimately realized their self-worth.

The teaching practicum exerted varying degrees and directions of influence on the core career motivations of the participants in this study. The reasons for these differences primarily include three points: Firstly, the duration of teaching practice. Zoe and Gimi had teaching practicum lasting for over a year, while Tina’s two teaching practicum each lasted for 3 months. This to some extent limited her ability to engage in deep reflection and adjustment. Secondly, the alignment of teaching practicum positions with their career aspirations. Despite Tina’s attempts at teaching practicum the alignment of the practice positions with international Chinese education was not high. This led her to believe that “her class was just for show and had no meaning.” Thirdly, the guidance provided during teaching practice. Although Gimi initially faced setbacks in his application for overseas practice, the guidance from his mentor helped him enhance his self-efficacy while feeling “recognized.” In contrast, Tina gradually lost confidence without any guidance.

### 5.4 Contribution of narrative inquiry to teacher motivation research

With the assistance of narrative inquiry as the methodology and ‘narrative thinking’ as the guiding principle, the current study has demonstrated the unique strengths and practicality of narrative inquiry in researching complex and dynamic construct such as teacher career motivation with its focus on narrative elements involving people, social relations, temporality, locality and so on to form the theoretical and actual backdrop. Xu et al. (2024) review the historical roots, developmental trajectory, and future outlooks of narrative inquiry in China, pointing out that narrative inquiry is a ‘relatively recent methodology’ (p. 2). Furthermore, they synthesize the application and understanding of narrative inquiry in different kinds of literature and demonstrate that research conducted in China has increasingly recognized the significance of narrative inquiry in capturing the complexities of educational contexts and the subjective experiences of educators within them. Therefore, situated in this academic landscape featuring rising interests in and popularity of this methodology, this research supplements the ongoing exploration of narrative inquiry in China with a new group of participants, expanding the contexts and depth where narrative inquiry is conducted. Therefore, against the backdrop of the academic landscape that witnesses a surge in interest and popularity of this methodology, the present research supports and promotes the ongoing investigation of narrative inquiry in China by introducing a fresh cohort of participants and new perspectives combining with metaphors. This addition not only broadens the scope of narrative inquiry, but also deepens its our understandings of pre-service CSL teacher motivation and the fundamental principles and prerequisites of the method itself that does justice to participant’s stories, narratives, and experiences.

### 5.5 Values of metaphors in narrative inquiry

As highlighted and exemplified by Craig (2018), novel and emergent metaphors that educators naturally hold merit situated examination that has personal and broader implications. In other words, the utilization of metaphors is helpful in understanding teachers’ knowing, doing, and being. Metaphors provide a symbolic and empirical language through which individuals make sense of their experiences, perceptions, and identities. In the context of pre-service teacher education, metaphors reveal teacher’s conceptualizations of teaching and learning, as well as their professional roles and identities in the making. The current study contributes to this discourse by exploring the metaphors employed by pre-service CSL teachers to articulate their professional identities and capture their developmental trajectories. On the basis of narrative inquiry, we have identified several illuminating metaphors used by Chinese teachers to describe their roles, challenges, and aspirations in the educational context, particularly throughout their experiences of teaching practicum. By highlighting these metaphors, this study sheds light on the nuanced ways in which pre-service CSL teachers perceive and navigate their professional journeys during and after the teaching practicum, one of the formative stages of their career perceptions and motivation, with agency. Moving forward, we intend to expand this research by delving deeper into teachers’ metaphors and their implications for teacher education. Specifically, we aim to explore how teachers’ metaphors influence their instructional approaches, classroom dynamics, and relationships with students, etc. Additionally, we plan to investigate the role of metaphors in shaping teachers’ professional development experiences and aspirations (Brandão, 2021).

As cautioned by Craig (2014, 2018), “stories to live by” could readily turn into “stories to leave by.” Therefore, it is imperative to implement policy reforms and other measures that foster conducive environments and conditions, as they are crucial for fostering sustainable “stories to live by” and avoiding the creation of or transformation into “stories to leave by.” That is to say, by conducting a comprehensive and sustained analysis of the personally experienced narratives of teachers, with a particular focus on their metaphors, across diverse and evolving contexts, future research holds the potential to offer profound insights that can facilitate teacher growth, augment teacher training programs, promote curriculum development efforts for MTCSOL, and steer educational policies and practices towards greater inclusivity and supportiveness.

## 6 Conclusion

The present study, through the means of narrative inquiry and the capture of metaphors, explores the developmental changes of pre-service CSL teachers’ career motivations in teaching practicum, and discusses the core driving forces behind their career choices and the influence on teaching practices. In this study, narrative inquiry reinforces how metaphors convey and shape participants’ feelings and reflections in teaching practice (Craig, 2013), illustrating the potential of metaphors in revealing the complexity of second language teachers’ identities (Pinho, 2019). The research findings indicate that participants’ career motivations undergo various dynamic changes, among which self-efficacy and intrinsic self-fulfillment motivation are the core motivational factors governing participants’ career choices. Furthermore, this study testifies to the significant influence of teaching practicum on teachers’ career motivations and the unique cross-cultural motivations of second language teachers from a longitudinal perspective. Therefore, the current study expands the depth of research on pre-service CSL teachers’ career motivations, helping us better understand the dynamic and complex factors behind pre-service CSL teachers’ decisions to enter or leave the profession, as well as their relevance to teaching practicum. In future research, other factors that might have an influence on career motivations, such as age, gender, previous experiences, educational resources and policies, significant others, etc., shall be integrated into the study of career motivations of CSL teachers. Given that metaphors are analytical tools with formative (as spaces for teacher reflection), experiential (as sources of teacher lived experiences and narratives), and analytical (as snapshots of teacher development) qualities (Brandão, 2021), future research should also continue to explore the unique role and value of metaphors as potent research tools, maintaining a consistent and focused approach.

## Data availability statement

The original contributions presented in the study are included in the article/supplementary material, further inquiries can be directed to the corresponding author/s.

## Ethics statement

The studies involving human participants were reviewed and approved by the ethics committee of the School of Chinese as a Second Language at Peking University. The studies were conducted in accordance with the local legislation and institutional requirements. Written informed consent to participate in this study was provided by all the participants. Written informed consent was obtained from the individuals for the publication of any potentially identifiable images or data included in this article.

## Author contributions

RZ: Writing – review & editing, Writing – original draft. TW: Writing – review & editing. HL: Writing – review & editing.
